# Anthracycline‐Induced Cardiomyopathy After Nephro‐/Neuroblastoma in Childhood: The Importance of Cardiological Reference Assessment

**DOI:** 10.1002/cam4.71158

**Published:** 2025-08-20

**Authors:** Kristina Kleen, Judith Gebauer, Claudia Spix, Lea L. Kronziel, Inke König, Katja Baust, Gabriele Calaminus, Thorsten Simon, Barbara Hero, Oliver Zolk, Norbert Graf, Hashim Abdul‐Khaliq, Thorsten Langer

**Affiliations:** ^1^ Paediatric Haematology and Oncology, Department of Paediatrics and Adolescent Medicine University Hospital Schleswig‐Holstein Lübeck Germany; ^2^ Medical Clinic 1 University Hospital Schleswig‐Holstein Lübeck Germany; ^3^ German Childhood Cancer Registry Mainz Germany; ^4^ Institute of Biometry and Statistics, University of Lübeck Lübeck Germany; ^5^ Paediatric Haematology and Oncology University Hospital Bonn Bonn Germany; ^6^ Paediatric Oncology and Haematology University Hospital Cologne Cologne Germany; ^7^ Institute for Clinical Pharmacology Immanuel Clinic Rüdersdorf, Brandenburg Medical School Rüdersdorf Germany; ^8^ Paediatric Haematology and Oncology University Hospital Homburg/Saar Homburg Germany; ^9^ Paediatric Cardiology University Hospital Homburg/Saar Homburg Germany

## Abstract

**Background:**

Long‐term childhood cancer survivors (CCS) may develop anthracycline‐induced cardiomyopathy. Our cross‐sectional study focused on the question of whether a central echocardiographic reference assessment is associated with a higher detection rate of cardiac dysfunction in a population‐based cohort of affected children with neuroblastoma or nephroblastoma. We also examined the prevalence of anthracycline‐induced cardiomyopathy and its risk factors.

**Methods and Patients:**

The cohort of this subproject comprises 370 nephroblastoma or neuroblastoma survivors diagnosed with cancer between 1990 and 2012. At study entry, participants were younger than 18 years old, had been treated with anthracyclines, and had no documented previous cardiac disease. Data were collected via patient questionnaires, cardiologic examinations in the network of adults with congenital heart defects (Erwachsene mit angeborenem Herzfehler [EMAH]) and a reference assessment of the recorded echocardiography.

**Results:**

The prevalence of cardiomyopathy in the study cohort (mean age: 12 years) was 6.3% at a median of 9.1 years after initial cancer diagnosis. Risk factors were an age under 5 years at tumor diagnosis and concomitant treatment with cyclophosphamide or radiation. As a central and novel finding, the detection rates by the EMAH cardiologists and the reference center are similarly high but discrepant.

**Discussion:**

Limitations were mainly due to the low responder rate and incomplete data. This study established a nationwide competence network linking pediatric oncology and cardiology centers across six university hospitals in Germany, enabling data collection on pediatric CCS. Despite lower case numbers compared to adult CCS cohorts, meaningful data were gathered and analyzed.

**Conclusion:**

Cardiac late effects after anthracycline‐based therapy in childhood affect a relevant proportion of long‐term CCS at pediatric age. In order to enable timely diagnosis and treatment, preventive examinations are essential and might benefit from additional central reference assessments. Discrepancy in detection of cardiomyopathy by reference and EMAH cardiologists requires further investigation.

## 1 Introduction

In Germany, approximately 2200 children and adolescents are diagnosed with cancer each year. Due to advances in diagnostics and multimodal therapies, the 15‐year survival rate has now exceeded 80% [1, 2]. However, more than two‐thirds of childhood cancer survivors (CCS) develop treatment‐related late effects [3]. Regular follow‐up care can facilitate early diagnosis of these complications and thus prevent hospitalizations [4].

Several risk factors for late effects have been identified, including the age at cancer treatment exposure. The embryonal tumors neuroblastoma and nephroblastoma/Wilms tumor typically occur in early childhood. Depending on tumor stage, chemotherapy with anthracyclines is an integral part of treatment for both malignancies [5]. These agents can cause acute and late myocardial damage through oxidative mechanisms and progressive remodeling, potentially leading to anthracycline‐induced cardiomyopathy, which may clinically manifest as heart failure [6, 7].

The prevalence of cardiomyopathy in the general pediatric population is 2.6 per 100,000 children (0.0026%) [8]. In CCS, the risk for cardiovascular diseases including cardiomyopathy, heart failure, and valvular dysfunction is up to eight times higher, making cardiovascular disease one of the leading causes of late mortality in this population [7]. Among CCS who have undergone anthracycline exposure, the risk of developing cardiomyopathy is increased 15 times [7, 9]. However, these agents are among the most effective anticancer drugs to date, and avoiding their use could negatively impact patient prognosis [7].

Cardiomyopathy may remain clinically asymptomatic for years before progressing to symptomatic heart failure [10, 11, 12, 13]. It is assumed that a progressive decline in left ventricular ejection fraction (LVEF), as assessed via echocardiography (echo), occurs, often in conjunction with additional cardiovascular risk factors [12]. Subclinical impairments of left ventricular systolic function can already be diagnosed through a reduced LVEF or fractional shortening [13] before CCS develop overt clinical disease. If treated early, these patients demonstrate a good functional recovery [7]. Echocardiographic screening can thus help to detect subclinical stages of cardiac dysfunction so that patients can benefit from specific therapeutic interventions [10, 11]. Among adult CCS, delayed diagnosis of cardiac dysfunction has been associated with a significantly reduced response to pharmacological therapy [10]. These findings highlight the importance of timely and effective cardiac screening in CCS. Current recommendations [10] advocate for lifelong echocardiographic monitoring.

While cardiac late effects occurring in adult CCS have been investigated in many studies, data on pediatric CCS are still limited. The subproject presented here as part of the study “Strukturoptimierung für krebskranke Kinder nach Anthrazyklintherapie. Eine Studie zur Ursache und Früherkennung der Anthrazyklin‐induzierten Kardiomyopathie nach Behandlung eines Nephro‐ und Neuroblastoms” (“Structural Optimization for Children with Cancer after Anthracycline Therapy: A Study on the Causes and Early Detection of Anthracycline‐Induced cardiomyopathy following Treatment for Nephroblastoma and Neuroblastoma”) [5] focusses on CCS under the age of 18 years and investigates whether the detection of cardiac dysfunction in this cohort is higher if performed by a central reference assessment than by one of the cardiologists in private practice organized in the network for adults with congenital heart defects (Erwachsene mit angeborenem Herzfehler [EMAH]; see also https://emah.dgk.org/zertifizierte‐emah‐zentren).

## 2 Methods

The German Childhood Cancer Registry (GCCR; Deutsches Kinderkrebsregister, DKKR) invited 2300 CCS to participate in the study who had been diagnosed with neuroblastoma or nephroblastoma between 1990 and 2012, had received anthracycline therapy according to the neuroblastoma and nephroblastoma study database, and were at least 5 years old at the time of cardiological follow‐up. Written consent was obtained from all participants. The study was based on three different assessments and included: (1) patient/parent questionnaires, (2) a cardiological examination (with echo/ECG), carried out by a cardiologist in private practice from the EMAH network in accordance with a Standard Operating Procedure (SOP), and (3) a reference assessment of the echo.

**FIGURE 1:**
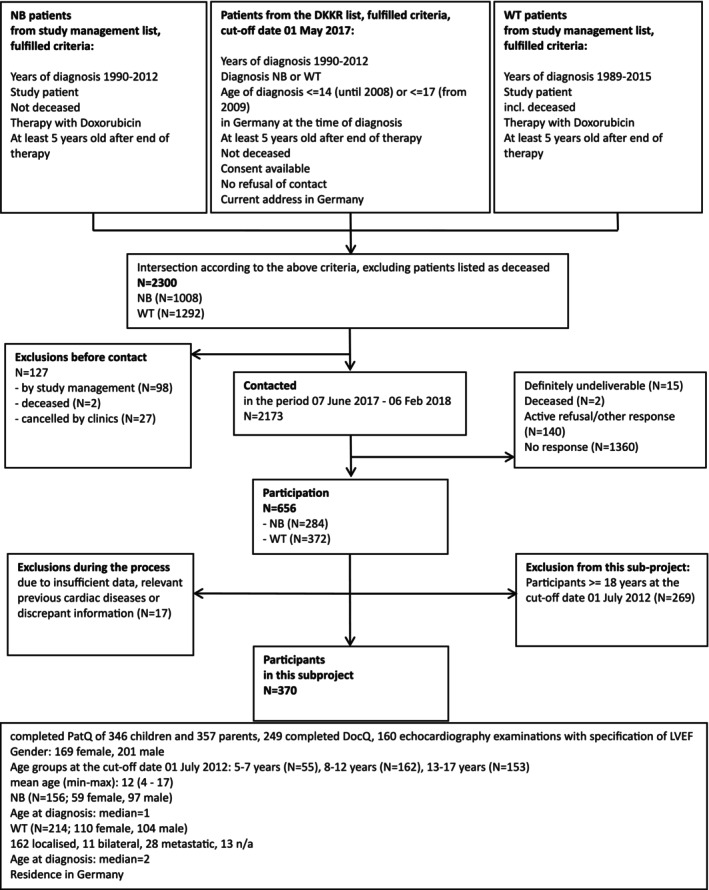
Illustration of the recruitment of study participants considering inclusion/exclusion criteria. DKKR, Deutsches Kinderkrebsregister, German Childhood Cancer Registry; LVEF, left ventricular ejection fraction; NB, neuroblastoma; WT, nephroblastoma/wilms tumor.

A total of 656 CCS, that is, 28.5% of the CCS contacted, were included in the study; of these, 370 CCS < 18 years of age at the cut‐off date July 01, 2012 were considered in this subproject (see Figure 1). Seven participants with (congenital) heart disease prior to tumor diagnosis remained included based on pre‐study cardiological assessments indicating no functional cardiac impairment. These were a ventricular septal defect, an atrial septal defect, a peripartum “hole in the heart that later closed”, a “small hole” and a mild leak in the aortic valve.

In 126/370 participants (34.1%), the cumulative anthracycline dose was not known. As only patients with documented anthracycline exposure were contacted to participate in the study, these CCS were included in this intention‐to‐treat analysis.

In this cross‐sectional study, the three different cardiac assessments (1–3) were carried out once during the study period. Data retrieved from follow‐up assessments were not included in this study.

Patient questionnaires (PatQ) on quality of life in the age‐specific subgroups 5–7, 8–12, and 13–17 years were used for CCS and their parents. The PatQ consisted of the pediatric quality of life questionnaire (PEDQOL) and the age‐adapted PedsQL 4.0. In addition, adolescent study participants (13–17 years) and the parents of the 5–12‐year‐old CCS were asked about physical activity, consumer behavior, illnesses, medication intake, and other information (including height, weight, leisure activities, cancer aftercare) in analogy to the KIGGS questionnaire of the Robert Koch Institute (RKI) (For further information, see “KiGGS‐Studie zur Gesundheit von Kindern und Jugendlichen in Deutschland”: https://www.rki.de/DE/Themen/Nichtuebertragbare‐Krankheiten/Studien‐und‐Surveillance/Studien/KiGGS/kiggs_start_inhalt.html).

CCS received a cardiological examination from cardiologists in private practice organized in the EMAH network. The cardiologists were asked to fill out a questionnaire (doctor's questionnaire (DocQ)) including information on height, weight, blood pressure, heart rate, subjective resilience, clinical findings, and cardiological diagnoses as well as echo/ECG results.

The recorded echo data sets were pseudonymised and sent to the German Paediatric Cardiology Reference Center in Homburg/Saar. Central reference evaluation was carried out by a single reference pediatric cardiologist blinded to the other clinical data to minimize interrater variability.

### 2.1 Data Analysis

Once data collection was completed, the data input was randomly checked for plausibility. The data from the PatQ, DocQ, and the echo data as well as data from the GCCR were analyzed together.

In some cases, several variables were available for one characteristic, for example, for weight and height from PatQ and DocQ. If deviations occurred, data were considered according to the following scheme: If both the doctor and the participant provided information on a characteristic, the participant's information (PatQ) was prioritized. If the participant did not provide any information on one or several characteristics, the doctor's information (DocQ) was used. Gender was obtained from data of the GCCR; in four cases, information on gender was retrieved from the PatQ due to missing data at the GCCR.

Implausible data (e.g., weight “43524.00”) were treated as missing. Clinical and disease/treatment‐specific data were supplemented from the data sets of the GCCR and the neuroblastoma and nephroblastoma study administrations.

Descriptive frequency distributions were created. Cross‐tabulations were used to determine the presence of a cardiomyopathy in subgroups with specific clinical characteristics or risk factors. Due to small numbers in the respective subgroups, statistical tests for group differences were not performed. SPSS version 27 was used to analyze the data.

### 2.2 Parameters for Cardiomyopathy

The EMAH cardiologists were asked to carry out a cardiological examination in accordance with a SOP provided by the study team. The echo was sent electronically to the reference center in Homburg/Saar. This center collected various echocardiological parameters in a blinded manner. The echocardiological diagnosis of cardiomyopathy was based on a reduction in LVEF to < 50% as proposed in the current guidelines of the European Society of Cardiology (ESC) [14], in the recommendations of the Delphi Panel Consensus 2022 [10] and in the Ross Score [15]. The LVEF values obtained are evaluated in the following as an assessment of the cardiological reference. Other echo parameters are not included in the reference assessment.

The assessment of the EMAH cardiologists was recorded in the DocQ based on the diagnosis of cardiomyopathy or heart failure. LVEF values were not included in the DocQ. If no diagnosis was given, this was not considered missing information, as we assumed that no cardiological diagnosis was made.

In summary, the *presence of cardiomyopathy* for the following analyses was therefore defined as an LVEF < 50% and/or a documented diagnosis of cardiomyopathy or heart failure in the DocQ.

To assess whether the detection of cardiac dysfunction is higher with a reference assessment, comparisons were made using a cross‐tabulation between the cardiological diagnoses of cardiomyopathy or heart failure in the DocQ (EMAH cardiologist) and the presence of cardiomyopathy according to LVEF (reference assessment) (see Tables 1 and 2). Due to a small number of cases of cardiomyopathies (*N* = 16), no statistical test for group differences could be performed.

**TABLE 1:** Comparison of CMP detections between EMAH cardiologists and reference assessment.

		LVEF (cut‐off for CMP < 50%)			LVEF missing	Total
		no CMP acc. to LVEF	CMP acc. to LVEF	Total		
DocQ completed	Missing	132	7	139	84	223
Cardiological diagnosis	**HF**	**2**	**0**	**2**	**0**	**2**
	aHT	4	0	4	5	9
	CA	4	0	4	4	8
	**CMP**	**7**	**0**	**7**	**0**	**7**
	Total	149	7	**156**	**93**	**249**
DocQ not completed		4	0	**4**	**117**	121
Total		153	7	**160**	210	**370**

*Note:* The assessment of the EMAH cardiologists was documented in the DocQ as CMP or HF, whereby the diagnosis of HF is considered a manifestation of CMP. The measured echocardiographic values of the LVEF indicate CMP from a cut‐off value of < 50% and are evaluated as an assessment of the cardiologic reference. LVEF specified, but no DocQ completed: 4/370; DocQ completed, but no LVEF specified: 93/370; both avaiable: 156/370; Total of 253 cases that can be included for the analysis of a CMP. 7x CMP according to DocQ, 2x HF according to DocQ, 7x CMP according to LVEF; Total of 16 cases with CMP. Red marks the cases with Heart Failures, Orange marks the cases with cardiomyopathy, and Blue marks all missing cases/cases with no information. Bold values mark numbers of interest.

Abbreviations: acc., according; aHT, arterial hypertension; CA, cardiac arrhythmia; CMP, cardiomyopathy; DocQ, doctor's questionnaire; EMAH, Erwachsene mit angeborenem Herzfehler; adults with congenital heart defect; EMAH, Erwachsene mit angeborenem Herzfehler; adults with congenital heart defect; HF, heart failure; LVEF, left ventricular ejection Fraction.

**TABLE 2:** Presentation of the cases with cardiomyopathy: Comparison of detections by EMAH cardiologists (DocQ) and reference (LVEF).

			DocQ completed: cardiological diagnosis					Total
			None	HF	aHT	CA	CMP	
LVEF (cut‐off for CMP < 50%)	No CMP acc. to LVEF	***N***	**0**	**2**	**0**	**0**	**7**	**9**
		% (LVEF)	0.00%	22.20%	0.00%	0.00%	77.80%	100.00%
		% (DocQ)	0.00%	100.00%	0.00%	0.00%	100.00%	56.30%
	CMP acc. to LVEF	***N***	**7**	**0**	**0**	**0**	**0**	**7**
		% (LVEF)	100.00%	0.00%	0.00%	0.00%	0.00%	100.00%
		% (DocQ)	100.00%	0.00%	0.00%	0.00%	0.00%	47.80%
	Total	***N***	**7**	**2**	**0**	**0**	**7**	**16**
		% (LVEF)	43.80%	12.50%	0.00%	0.00%	43.80%	100.00%
		% (DocQ)	100.00%	100.00%	0.00%	0.00%	100.00%	100.00%

*Note:* The assessment of the EMAH cardiologists was documented in the DocQ as CMP or HF, whereby the diagnosis of HF is considered a manifestation of CMP. The measured echocardiographic values of the LVEF indicate CMP from a cut‐off value of < 50% and are evaluated as an assessment of the cardiologic reference. A total of 16 cardiomyopathies were detected. In the rows, the echo parameter LVEF (reference) indicates whether a cardiomyopathy is present. The columns show the cardiological diagnoses (EMAH), with CMP and HF as manifestations of CMP indicating the presence of a cardiomyopathy. Red marks the cases with Heart Failures, Orange marks the cases with cardiomyopathy. Bold values mark numbers of interest.

Abbreviations: acc., according; aHT, arterial Hypertension; CA, cardiac arrhythmia; CMP, cardiomyopathy; DocQ, doctor's questionnaire; EMAH, Erwachsene mit angeborenem Herzfehler; adults with congenital heart defect; HF, heart failure; LVEF, left ventricular ejection fraction.

In addition, the presence of cardiomyopathy was compared with the participants' statements in the PatQ as to whether heart failure/cardiomyopathy had ever been reported (see Tables 3 and 4).

**TABLE 3:** Comparison of LVEF (reference) with information from the PatQ on “Has a doctor or carer ever told you that you have heart failure?”

			PatQ: Has a doctor or carer ever told you …				Total
			Missing	Yes	No	Don't know	
LVEF	Missing	***N***	**10**	**5**	**190**	**5**	**210**
		% (LVEF)	4.80%	2.40%	90.50%	2.40%	100.00%
		% (PatQ)	62.50%	62.50%	56.70%	45.50%	56.80%
	No CMP	***N***	**6**	**2**	**139**	**6**	**153**
		% (LVEF)	3.90%	1.30%	90.80%	3.90%	100.00%
		% (PatQ)	37.50%	25.00%	41.50%	54.50%	41.40%
	CMP	***N***	**0**	**1**	**6**	**0**	**7**
		% (LVEF)	0.00%	14.30%	85.70%	0.00%	100.00%
		% (PatQ)	0.00%	12.50%	1.80%	0.00%	1.90%
Total		***N***	**16**	**8**	**335**	**11**	**370**
		% (LVEF)	4.30%	2.20%	90.50%	3.00%	100.00%
		% (PatQ)	100.00%	100.00%	100.00%	100.00%	100.00%

*Note:* CMP is present with LVEF < 50%. Bold values mark numbers of interest.

Abbreviations: CMP, cardiomyopathy; LVEF, left ventricular ejection fraction; PatQ, patient's questionnaire.

**TABLE 4:** Comparison of the cardiological diagnosis from the DocQ (EMAH) with information from the PatQ on “Has a doctor or carer ever told you that you have heart failure?”

			PatQ: Has a doctor or carer ever told you…				Total
			Missing	Yes	No	Don't know	
Cardiological diagnosis (DocQ)	Missing	***N***	**15**	**7**	**313**	**9**	**344**
		% (cardiological diagnosis)	4.40%	2.00%	91.00%	2.60%	100.00%
		% (PatQ)	93.80%	87.50%	93.40%	81.80%	93.00%
	HF	***N***	**0**	**0**	**1**	**1**	**2**
		% (cardiological diagnosis)	0.00%	0.00%	50.00%	50.00%	100.00%
		% (PatQ)	0.00%	0.00%	0.30%	9.10%	0.50%
	aHT	***N***	**0**	**0**	**9**	**0**	**9**
		% (cardiological diagnosis)	0.00%	0.00%	100.00%	0.00%	100.00%
		% (PatQ)	0.00%	0.00%	2.70%	0.00%	2.40%
	CA	***N***	**0**	**0**	**7**	**1**	**8**
		% (cardiological diagnosis)	0.00%	0.00%	87.50%	12.50%	100.00%
		% (PatQ)	0.00%	0.00%	2.10%	9.10%	2.20%
	CMP	***N***	**1**	**1**	**5**	**0**	**7**
		% (cardiological diagnosis)	14.30%	14.30%	71.40%	0.00%	100.00%
		% (PatQ)	6.30%	12.50%	1.50%	0.00%	1.90%
Total		***N***	**16**	**8**	**335**	**11**	**370**
		% (cardiological diagnosis)	4.30%	2.20%	90.50%	3.00%	100.00%
		% (PatQ)	100.00%	100.00%	100.00%	100.00%	100.00%

*Note:* According to EMAH cardiologists, CMP is present in the case of cardiological diagnoses of CMP or HF as a manifestation of CMP. Bold values mark numbers of interest.

Abbreviations: aHT, arterial hypertension; CA, cardiac arrhythmia; CMP, cardiomyopathy; DocQ, doctor's questionnaire; EMAH, Erwachsene mit angeborenem Herzfehler; adults with congenital heart defect; HF, heart failure; PatQ, patient's questionnaire.

### 2.3 Risk Factors for the Development of Cardiac Late Effects

The following risk factors for the development of late cardiac events were analyzed using cross‐tabulations: Time since initial diagnosis [9], younger age at diagnosis [16], female gender [17, 18], mediastinal or pulmonary irradiation > 15 Gy [9, 19], concurrent therapy with cyclophosphamide [18], the presence of arterial hypertension [11] and a cumulative anthracycline dose > 250 mg/m^2^, which in combination with mediastinal or pulmonary irradiation further potentiates the risk [17, 19]. These and the two tumor entities were compared with the presence of cardiomyopathy (see Table 5).

**TABLE 5:** The presence of cardiomyopathy after treatment with anthracyclines in CCS of neuro‐/nephroblastoma aged < 18 year.

			CMP is present if A		Total
			CMP not present	CMP present	
Tumor	Nephroblastoma	***N***	**139**	**9**	**148**
		% (Tumor)	93.9%	6.1%	100.0%
		% (CMP is present if A)	58.6%	56.3%	58.5%
	Neuroblastoma	***N***	**98**	**7**	**105**
		% (Tumor)	93.3%	6.7%	100.0%
		% (CMP is present if A)	41.4%	43.8%	41.5%
	Total	***N***	**237**	**16**	**253**
		% (Tumor)	93.7%	6.3%	100.0%
		% (CMP is present if A)	100.0%	100.0%	100.0%
Gender	Female	***N***	**110**	**7**	**117**
		% (Gender)	94.0%	6.0%	100.0%
		% (CMP is present if A)	46.4%	43.8%	46.2%
	Male	***N***	**127**	**9**	**136**
		% (Gender)	93.4%	6.6%	100.0%
		% (CMP is present if A)	53.6%	56.3%	53.8%
	Total	***N***	**237**	**16**	**253**
		% (Gender)	93.7%	6.3%	100.0%
		% (CMP is present if A)	100.0%	100.0%	100.0%
Age at diagnosis (years)	Present	*N*	237	16	253
		**Median**	**1**	**1**	**1**
		(min–max)	(0–8)	(0–5)	(0–8)
	Missing	*N*	0	0	0
Time since initial diagnosis (years)	Present	*N*	225	16	241
		**Median**	**10.2**	**9.1**	**10**
		(min–max)	(4.8‐17.6)	(5.5‐16.4)	(4.8‐17.6)
	Missing	*N*	12	0	12
Cumulative anthracycline dose(mg/m^2^)	< 250.00 without 0.00	***N***	**120**	**10**	**130**
		% (cumulative anthracycline dose)	92.3%	7.7%	100.0%
		% (CMP is present if A)	50.6%	62.5%	51.4%
	≥ 250.00	***N***	**32**	**2**	**34**
		% (cumulative anthracycline dose)	94.1%	5.9%	100.0%
		% (CMP is present if A)	13.5%	12.5%	13.4%
	0 (missing)	***N***	**85**	**4**	**89**
		% (cumulative anthracycline dose)	95.5%	4.5%	100.0%
		% (CMP is present if A)	35.9%	25.0%	35.2%
	Total	***N***	**237**	**16**	**253**
		% (cumulative anthracycline dose)	93.7%	6.3%	100.0%
		% (CMP is present if A)	100.0%	100.0%	100.0%
Cumulative anthracycline dose(mg/m^2^)	> 0.00	*N*	152	12	164
		**Median**	**180**	**180**	**180**
		(min–max)	(26–300)	(53–350)	(26–350)
	0 (missing)	*N*	85	4	89
Other potentially cardiotoxic drugs	None	***N***	**129**	**8**	**137**
		% (other potentially cardiotoxic drugs)	94.2%	5.8%	100.0%
		% (CMP is present if A)	54.4%	50.0%	54.2%
	Cyclophosphamide	***N***	**10**	**1**	**11**
		% (other potentially cardiotoxic drugs)	90.9%	9.1%	100.0%
		% (CMP is present if A)	4.2%	6.3%	4.3%
	Missing	***N***	**98**	**7**	**105**
		% (other potentially cardiotoxic drugs)	93.3%	6.7%	100.0%
		% (CMP is present if A)	41.4%	43.7%	41.5%
	Total	***N***	**237**	**16**	**253**
		% (other potentially cardiotoxic drugs)	93.7%	6.3%	100.0%
		% (CMP is present if A)	100.0%	100.0%	100.0%
External irradiation	Yes	***N***	**17**	**2**	**19**
		% (external irradiation)	89.5%	10.5%	100.0%
		% (CMP is present if A)	7.2%	12.5%	7.5%
	No	***N***	**122**	**7**	**129**
		% (external irradiation)	94.6%	5.4%	100.0%
		% (CMP is present if A)	51.5%	43.8%	51.0%
	Unknown	***N***	**3**	**0**	**3**
		% (external irradiation)	100.0%	0.0%	100.0%
		% (CMP is present if A)	1.2%	0.0%	1.2%
	Missing	***N***	**95**	**7**	**102**
		% (external irradiation)	93.1%	6.9%	100.0%
		% (CMP is present if A)	40.1%	43.8%	40.3%
	Total	***N***	**237**	**16**	**253**
		% (external irradiation)	93.7%	6.3%	100.0%
		% (CMP is present if A)	100.0%	100.0%	100.0%
Arterial Hypertension(PatQ)	Yes	***N***	**24**	**3**	**27**
		% (arterial Hypertension)	88.9%	11.1%	100.0%
		% (CMP is present if A)	10.1%	18.8%	10.7%
	No	***N***	**194**	**11**	**205**
		% (arterial Hypertension)	94.6%	5.4%	100.0%
		% (CMP is present if A)	81.9%	68.8%	81.0%
	Unknown	***N***	**5**	**1**	**6**
		% (arterial Hypertension)	83.3%	16.7%	100.0%
		% (CMP is present if A)	2.1%	6.3%	2.4%
	Missing	***N***	**14**	**1**	**15**
		% (arterial Hypertension)	93.3%	6.7%	100.0%
		% (CMP is present if A)	5.9%	6.3%	5.9%
	Total	***N***	**237**	**16**	**253**
		% (arterial Hypertension)	93.7%	6.3%	100.0%
		% (CMP is present if A)	100.0%	100.0%	100.0%

*Note:* Distribution according to tumor entities and risk factors. A = LVEF < 50% and/or documented diagnosis of cardiomyopathy or heart failure in the DocQ. Bold values mark numbers of interest.

Abbreviations: CCS, childhood cancer survivor; CMP, cardiomyopathy; DocQ, doctor's questionnaire; LVEF, left ventricular ejection fraction; PatQ, patient's questionnaire.

### 2.4 Ethics approval statement

The study was evaluated positively by the ethics committees of the University Erlangen‐Nuremberg (reference 3750) and of the University of Lübeck (reference 14‐182). These ethics committees both provided approval as Thorsten Langer, who developed this study at the University Erlangen‐Nuremberg in 2012, moved to the University of Lübeck in 2013.

## 3 Results

### 3.1 Data on Cardiomyopathy

In the present study, the *overall prevalence of cardiomyopathy* in this cohort was 6.3% (16/253) after a median time since initial diagnosis of 9.1 years (min–max = 5.5–16.4 years) (see Table 5).

For 93 participants, a DocQ was completed but LVEF parameters from the reference center were missing. In four cases, LVEF values from the reference center were available, but no completed DocQ. For a further 156 participants, both a completed DocQ and an LVEF were available. Thus, a total of 253/370 cases (68.4%) could be included in the analysis of late cardiomyopathy (see Table 1).

Table 1 provides a comparison of the cardiomyopathy rates according to LVEF (echo/reference assessment) and cardiological diagnosis documented in the DocQ (EMAH cardiologists) in order to assess whether the detection rate of cardiac dysfunction was higher if performed by a central reference assessment than by cardiologists in private practice.

In detail, a *DocQ* was completed in 249/370 cases (67.3%). *Cardiological diagnoses* (heart failure, arterial hypertension, cardiac arrhythmia, cardiomyopathy) were reported in 26 cases, of which 2/249 were heart failure (0.8%, each NYHA1 (class I according to the New York Heart Association)) and 7/249 cardiomyopathy (2.8%). For all nine cases with documented heart failure or cardiomyopathy in the DocQ, normal LVEF values without evidence of cardiomyopathy were recorded in the reference center.

In the cardiology *reference* center, the *echo parameter LVEF* was analyzed in 160/370 cases (43.2%). 7/160 cases (4.4%) fulfilled the diagnostic criterion of cardiomyopathy with an LVEF < 50%. In all seven cases, no cardiological diagnosis/no cardiomyopathy was stated in the DocQ. Further investigations of these cases were not possible due to data protection regulations.

In summary, there was no concordance between the 9 and 7 cases with cardiomyopathy diagnosed either by the EMAH cardiologists or the cardiology reference center, respectively (see Tables 1 and 2).

When considered separately, the detection rate by the EMAH cardiologists was 3.6% (9/249) and by the reference center 4.4% (7/160) (see Table 1).

In the *PatQ*, 8/354 (2.3%) of the study participants indicated that they had been diagnosed with heart failure. In one of these cases (1/8, 12.5%), diagnosis was confirmed by the reference center (see Table 3); in another case (12.5%) by the DocQ (EMAH cardiologists) (see Table 4). All of the other cases (6/8, 75%) could be confirmed neither by a cardiological diagnosis from the EMAH cardiologists nor by a reduced LVEF value. However, for five of those eight cases, no LVEF value was available.

12/370 CCS (3.2%) stated they were receiving medical treatment for their heart disease. Among the 16 cases of cardiomyopathy, candesartan was reported in one case in order to treat hypertension; any further cardioprotective medication was not documented. Furthermore, 208/370 CCS (56.2%) stated that they had taken any medication in the last 7 days. Almost 9% of CCS (33/370) reported medication with l‐thyroxine; however, information on MIBG therapy as a potential risk factor for hypothyroidism was not available to further investigate this observation.

### 3.2 Risk Factors

The *cumulative anthracycline dose* was reported in 244/370 cases (66%) (median [min–max] = 180 [17–350] mg/m^2^, 192/244 (78.7%) < 250 mg/m^2^, 52/244 (21.3%) ≥ 250 mg/m^2^).

Information on the variable “other potentially cardiotoxic drugs” was provided in 214/370 cases (57.8%), with 17/214 (7.9%) additionally treated with *cyclophosphamide*.

In 212/370 cases (57.3%), information on *external radiotherapy* (RT) was available. All cases were nephroblastoma survivors. 31/212 (14.6%) were exposed to RT (8/31 (25.8% each) right or left abdominal RT, 6/31 (19.4%) total abdominal and 9/31 (29%) chest RT). The cumulative radiation dose was below 15 Gy in all cases.

Table 5 presents therapy‐associated risk factors and other risk factors (e.g., gender and age at tumor diagnosis) in CCS with cardiomyopathy.

### 3.3 Follow‐Up Care

289/346 (83.5%) of CCS attended follow‐up appointments (218/289 (75.4%), 1–2 times per year); 57/289 (16.5%) no longer attended follow‐up. 244/289 (84.4%) of the CCS went to their pediatric oncology department, 24/289 (8.3%) to their family doctor/pediatrician, and 21/289 (7.3%) visited specialized long‐term follow‐up clinics.

## 4 Discussion

The majority of CCS is affected by late effects occurring years to decades after the end of cancer therapy. Treatment with anthracyclines increases the risk for cardiotoxic damage, consecutive cardiomyopathy, and clinical heart failure [3, 5, 7]. In the present study, neuroblastoma and nephroblastoma survivors were questioned and examined to determine the presence of heart failure or cardiomyopathy. Moreover, detection of cardiomyopathy was compared between cardiologists in private practice and a national reference center to evaluate different approaches in long‐term follow‐up care. As lifelong cardiological monitoring is recommended for CCS at‐risk, and early diagnosis facilitates treatment of late cardiac effects, optimized surveillance strategies are essential to improve long‐term outcomes.

In order to compare the detection of cardiomyopathy between the two groups, EMAH cardiologists and the reference center, the echo parameter LVEF and the cardiological diagnoses documented in the DocQ were analyzed. The prevalence of cardiomyopathy was 6.3% after a median time of 9.1 years. This prevalence is elevated compared to an age‐adjusted general population, but in line with previous studies performed in cancer survivors who were exposed to cardiotoxic treatment [8]. Van der Pal et al. [16], for example, showed a prevalence of 27% for asymptomatic heart failure in adult CCS exposed to cardiotoxic therapy 15 years after cancer diagnosis. The reference evaluation in our study enabled us to include subclinical cases with reduced LVEF as well, which might result in a more realistic assessment of both clinically relevant as well as subclinical forms of late cardiotoxicity. However, on the other hand, it should be considered that this approach may have also caused a potential overestimation.

Notably, no diagnosis of cardiomyopathy or heart failure provided by EMAH cardiologists in private practice (DocQ) was confirmed by a reduced LVEF < 50% in the reference cardiological assessment and vice versa. This may be due to stabilized cardiac function under appropriate medication, so that the documented cardiomyopathy on the DocQ corresponded to a successfully treated cardiomyopathy. Studies have shown a good recovery from anthracycline‐induced cardiac dysfunction if it was diagnosed and treated early [7]: In patients with an LVEF ≤ 45% after anthracycline therapy, recovery of LVEF was observed in 42% of cases with enalapril and carvedilol treatment [7]. Comparing these results to the patient's questionnaire (PatQ), the majority of CCS affected by cardiomyopathy stated that they had never been diagnosed with this condition. However, one of these CCS reported medical treatment for heart diseases. As adherence to long‐term follow‐up was 83.5% in our cohort, lack of routine cardiological examinations is unlikely. However, awareness for late effects of cancer treatment might still need improvement in order to provide adequate information for both survivors and health care professionals. As adolescent participants (13–17 years) were asked to complete this part of the PatQ without parental help, this discrepancy may also be due to a reduced relevance of the disease for the adolescents.

Another reason for the discrepancies could be that subclinical stages diagnosed by the EMAH cardiologists might not have been communicated regularly. According to Armenian et al. [13], there is often a long latency between potentially cardiotoxic exposure and clinically symptomatic manifestation of cardiomyopathy in CCS. The asymptomatic period is characterized by echo parameters such as a thinned wall, a widening of the diameter, and subsequent increase in wall tension of the left ventricle. These subclinical changes may lead to impaired left ventricular systolic function and manifest as reduced LVEF or fractional shortening over time. In the present study, only LVEF was considered for the reference assessment. Future studies might focus on further echo parameters for the reference assessment that could also indicate an early stage of cardiomyopathy.

Importantly, the reference center only used LVEF < 50% as a criterion to diagnose cardiomyopathy, while EMAH cardiologists may have used other echo parameters and clinical criteria, which might explain some of the discrepancies documented between the two approaches.

However, missing a reduced LVEF < 50% by the EMAH cardiologists cannot be fully justified.

When assessing the discrepancies, it should also be considered that the reference assessment was performed by a single person to minimize interrater variability, while many EMAH cardiologists were involved; this may lead to a larger variance.

If the detection rate of asymptomatic/subclinical cardiac dysfunction in particular is higher by a reference assessment than by the local EMAH cardiologists, it cannot be answered adequately at this point. However, it can be assumed that, in addition to follow‐up care provided by specialized pediatric cardiologists, such as those at EMAH, a reference assessment might be useful to diagnose as many late cardiac complications as early as possible through various expert opinions and to facilitate timely therapy. As adherence to follow‐up appointments was high in our cohort, it can be assumed that many CCS exposed to anthracycline therapy are interested in surveillance for late effects.

Experts disagree on whether cardiological screening should be started within the first year after the end of treatment and on the frequency of screening [20] in the group of < 18‐year‐olds. The increasing time since initial diagnosis represents a risk factor for late cardiac complications [9]. However, in our analysis, we could demonstrate one survivor with a diagnosis of cardiomyopathy already 5 years after tumor diagnosis. It could therefore be assumed that follow‐up screening should start no later than 5 years after tumor diagnosis.

### 4.1 Limitations and Strengths of the Study

The present study succeeded in linking several competences in pediatric hematology and oncology as well as pediatric cardiology at the University Hospitals in Lübeck, Mainz, Saarland, Brandenburg, Bonn, and Cologne to form a competence network for congenital heart defects and to collect data throughout Germany. Compared to cases of adult CCS, the smaller number of cases of pediatric CCS could be collected and evaluated.

The main limitations were due to the partially incomplete or missing data. For example, LVEF values were only provided in 43.2% of cases and other echocardiographic values, such as the global longitudinal strain (GLS), which could have been included in the assessment of cardiomyopathy, were missing. Other parameters, especially N‐terminal pro brain natriuretic peptide (NT‐proBNP), were not recorded, but might have indicated, if elevated, an increased risk of developing cardiomyopathy with normal LVEF [21]. In the end, only 68.4% of the participants could be included in the analyses of cardiomyopathy, as neither LVEF values nor a completed medical questionnaire were available for the remaining cases. The small number of cardiomyopathies (*N* = 16) also limited the use of statistical tests. Consequently, the comparison of detection rates and the determination of risk factors for cardiomyopathy could only be analyzed descriptively. With regard to risk factors, data on radiotherapy and cyclophosphamide therapy for the neuroblastoma survivors were not provided and anthracycline dose was only known for 66% of cases in general. We also did not have sufficient data on any cardioprotective therapy, which might prevent the manifestation of cardiomyopathy in some cases [21] and could provide further explanations for the discrepancies in the detections between the EMAH cardiologist and the reference assessment.

Another limiting factor is the single date of examination by the EMAH cardiologists, which was used to determine the “time since initial diagnosis.” Firstly, this made it impossible to assess progression. Secondly, the criteria for cardiomyopathy could have been present at an earlier point in time, which would have influenced the risk factor “time since initial diagnosis/therapy.” At this point, a falsification of the time variable cannot be excluded.

The low participation rate of 28.5% of the CCS contacted may also have resulted in a non‐responder bias. Even if the analysis of a total of 370 CCS < 18 years can be seen as one of the strengths of these studies and the non‐responder analysis showed no relevant differences, a possible bias in the study results is possible.

Finally, the relatively short observation period of a median of 10 years after tumor diagnosis can be considered too short, as cardiac late effects may not yet have developed at this time or could not yet be detected by LVEF. On the other hand, an increased prevalence of cardiomyopathies was already detected after this relatively short period, which emphasizes the relevance of early cardiological screening.

## 5 Conclusion

In the present study, we could demonstrate an increased prevalence of cardiomyopathies after cardiotoxic cancer therapy already present in a young cohort of neuroblastoma and nephroblastoma survivors under the age of 18 years. While the association between anthracyclines and cardiomyopathy is well established, our study provides a key novel finding by highlighting diagnostic discrepancies between clinical and reference assessments in a pediatric cohort that has not yet been extensively studied. This could provide indications for the standardization of cardiological follow‐up care, which could benefit from an additional reference assessment.

Despite the use of standardized diagnostic procedures and clearly defined assessment criteria, subjective assessment differences are more likely to occur in decentralized examinations. These can be minimized by a reference assessment, thus optimizing the comparability and validity of the diagnostic results, which is particularly important for multicenter studies or registries.

For follow‐up studies, it would be interesting to further investigate the discrepancies in diagnosing cardiomyopathy between cardiologists in private practice and reference assessments. In addition, further echocardiographic values such as wall thickness and GLS as well as biomarkers such as NT‐proBNP should be included in the reference assessment.

In summary, the present results extend the findings previously related to adult CCS with additional findings from a markedly younger population and thus provide potentially relevant information for the organization of cardiological follow‐up care for young CCS.

## Author Contributions

**Kristina Kleen:** investigation, formal analysis, writing – original draft, writing – review and editing. **Judith Gebauer:** investigation, formal analysis, writing – original draft, writing – review and editing. **Claudia Spix:** conceptualization, investigation, formal analysis, writing – review and editing, data curation, methodology, software. **Lea L. Kronziel:** formal analysis, writing – review and editing, data curation, methodology. **Inke König:** formal analysis, writing – review and editing, data curation, methodology. **Katja Baust:** conceptualization, investigation, formal analysis, writing – review and editing. **Gabriele Calaminus:** conceptualization, investigation, formal analysis, writing – review and editing, funding acquisition. **Thorsten Simon:** conceptualization, investigation, formal analysis, writing – review and editing, funding acquisition, data curation, methodology, resources. **Barbara Hero:** conceptualization, investigation, formal analysis, writing – review and editing, data curation, methodology, resources. **Oliver Zolk:** conceptualization, investigation, formal analysis, writing – review and editing, data curation, methodology, software. **Norbert Graf:** conceptualization, investigation, formal analysis, writing – original draft, writing – review and editing, funding acquisition, data curation, methodology, resources, software. **Hashim Abdul‐Khaliq:** conceptualization, investigation, formal analysis, writing – review and editing, funding acquisition. **Thorsten Langer:** conceptualization, investigation, formal analysis, writing – original draft, writing – review and editing, funding acquisition, project administration.

## Ethics Statement

The study was evaluated positively by the ethics committees of the University of Erlangen‐Nuremberg (reference 3750) and the University of Lübeck (reference 14‐182).

## Conflicts of Interest

The authors declare no conflicts of interest.

## Data Availability

The data that support the findings of this study are available from the corresponding author upon reasonable request.
